# Phylogenetic analysis of the SAP30 family of transcriptional regulators reveals functional divergence in the domain that binds the nuclear matrix

**DOI:** 10.1186/1471-2148-9-149

**Published:** 2009-06-30

**Authors:** Keijo M Viiri, Taisto YK Heinonen, Markku Mäki, Olli Lohi

**Affiliations:** 1 Paediatric Research Centre, University of Tampere Medical School and Tampere University Hospital, 33520 Tampere, Finland

## Abstract

**Background:**

Deacetylation of histones plays a fundamental role in gene silencing, and this is mediated by a corepressor complex containing Sin3 as an essential scaffold protein. In this report we examine the evolution of two proteins in this complex, the Sin3-associated proteins SAP30L and SAP30, by using an archive of protein sequences from 62 species.

**Results:**

Our analysis indicates that in tetrapods SAP30L is more similar than SAP30 to the ancestral protein, and the two copies in this group originated by gene duplication which occurred after the divergence of Actinopterygii and Sarcopterygii about 450 million years ago (Mya). The phylogenetic analysis and biochemical experiments suggest that SAP30 has diverged functionally from the ancestral SAP30L by accumulating mutations that have caused attenuation of one of the original functions, association with the nuclear matrix. This function is mediated by a nuclear matrix association sequence, which consists of a conserved motif in the C-terminus and the adjacent nucleolar localization signal (NoLS).

**Conclusion:**

These results add further insight into the evolution and function of proteins of the SAP30 family, which share many characteristic with nuclear scaffolding proteins that are intimately involved in regulation of gene expression. Furthermore, SAP30L seems essential to eukaryotic biology, as it is found in animals, plants, fungi, as well as some taxa of unicellular eukaryotes.

## Background

The Sin3 multiprotein complex plays a central role in gene silencing by deacetylating histones, and deletion of the mouse SIN3A gene results in lethality at a postimplantation stage of development [1]. SAP30 was initially found in *Saccharomyces cerevisiae *and human cells as a protein which co-immunopurified with the Sin3 corepressor complex [2-4]. In cultured cells, SAP30 is not necessary for repression activity by the Sin3 complex, but it participates in N-CoR-mediated repression by specific transcription factors [4]. Thus, it functions as a bridging and stabilizing molecule between the Sin3 complex and corepressors such as N-CoR [4] and CIR [5], and DNA-binding transcription factors such as YY1 [6]. Mammals have one paralog of SAP30, named "SAP30-like" (SAP30L), which shares 70% sequence identity with SAP30 [7]. SAP30L also binds to the Sin3A complex and represses transcription when tethered to different promoters [8]. In *S. cerevisiae*, SAP30 has been shown to be involved in regulation of transcription of the HMR, telomeric, and rDNA loci [9,10], and SAP30-deficient yeast strains have defects in ribosomal rRNA processing [11]. Consistent with a nucleolar function, we have previously identified nucleolar localization signals (NoLSs) in human SAP30 and SAP30L, and showed that they can direct Sin3A to the nucleolus [8].

Recently, we identified by mass spectrometric studies a C2CH-type zinc-binding module in the N-termini of SAP30 and SAP30L [12]. An independent NMR-study also confirmed the results that SAP30 proteins contain C2CH-type large zinc fingers [13]. This structure is essential for the stability and DNA-binding activity of both proteins. Close to the zinc-binding module resides a polybasic region originally identified as a nuclear localization signal (NLS) in SAP30L [7]. We showed that this region, together with the preceding hydrophobic region, mediates specific interactions of SAP30/SAP30L with the monophosphoinositides (PIPs) PdtIns3P, PtdIns4P and PtdIns5P. Intriguingly, DNA- and PIP-binding occur in the same region and compete with each other. Increasing the concentration of monophosphosphoinositides leads to the release of DNA from SAP30/SAP30L, and reduced transcriptional repression [12]. Furthermore, we showed that SAP30 and SAP30L interact with core histones and nucleosomes and that this interaction is partly mediated by the central acidic region [12].

Nucleotide sequences belonging to the SAP30 family have been found in many eukaryotic species, but most of these putative homologs remain unrecognized and uncharacterized in databases, including those of the NCBI. Here we present a phylogenetic analysis of proteins of the SAP30 family. Our analyses indicate that SAP30L is the ancestral protein of this family and it is found in animals, plants, fungi and some protists. A single duplication event of an ancient SAP30L-bearing chromosome segment gave rise to the SAP30 gene. The most conserved region in SAP30 proteins is in the C-terminus, and we show by biochemical experiments that this region is responsible for association with the nuclear matrix. Phylogenetic analysis reveals that SAP30 has accumulated mutations in its C-terminus, and this has reduced its association with the nuclear matrix. This study suggests that proteins of the SAP30 family play a role in Sin3-mediated repression through multiple interactions with the nuclear matrix, nuclear proteins and DNA.

## Results and discussion

### SAP30 and SAP30L genes in the human, mouse, chicken and zebrafish genomes

**Figure 1:**
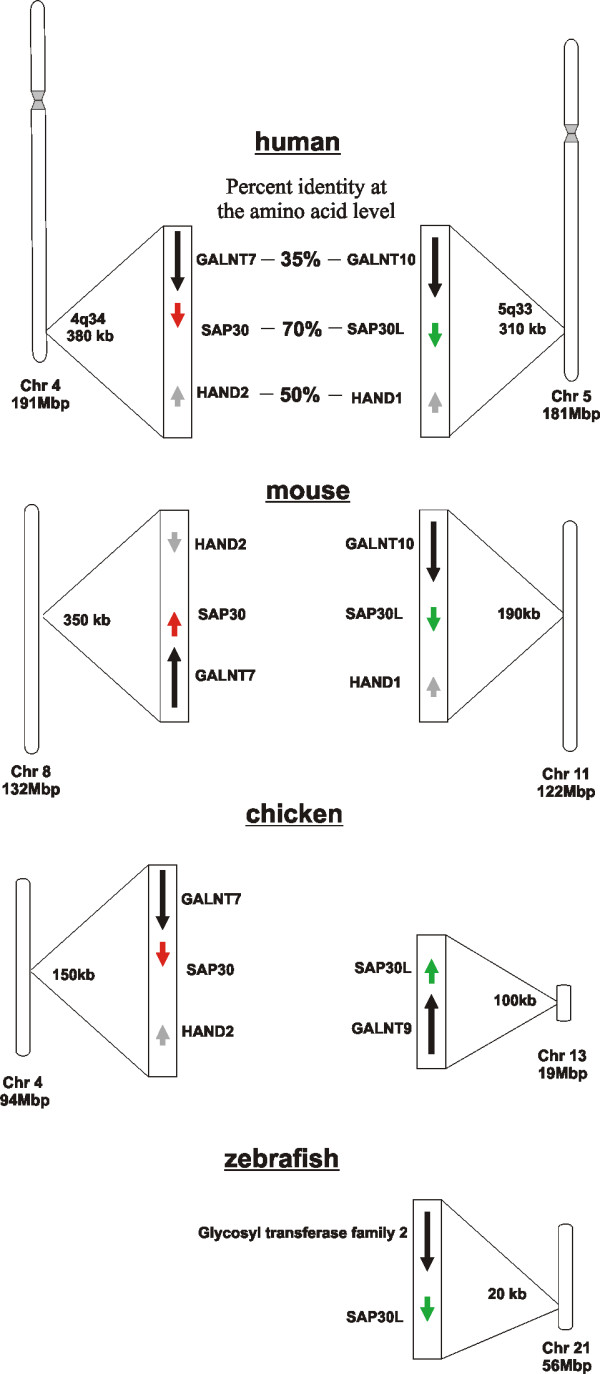
**Chromosomal localizations of the SAP30 family genes in the *Homo sapiens, Mus musculus, Gallus gallus *and *Danio rerio *genomes**. The chromosome number and the approximate length (in Mb) are indicated below each chromosome. The lengths of the syntenies are presented in kb. The degree of similarity in the derived amino acid sequence in the Clustal V alignment is indicated as a percentage of identical residues for the human genes.

The human SAP30 and SAP30L genes are located in chromosome bands 4q34.1 and 5q33.2, respectively (Figure 1). Careful analyses reveal that similar genes flank the SAP30 and SAP30L genes in their respective chromosomes and, in fact, these two chromosomes are known to share duplicated segments [14]. The GALNT 10 and GALNT7 genes are located upstream of the SAP30L and SAP30 genes, respectively. On the downstream side, the SAP30L gene is followed by HAND1, and the SAP30 gene by HAND2. It is noteworthy that the degree of sequence identity between the proteins encoded by these flanking genes is similar to that between SAP30 and SAP30L, when aligned by Clustal V. Since the occurrence of a chromosomal duplication event seemed likely, we analyzed other organisms for the presence of this GALNT-SAP-HAND block in order to estimate the time of the duplication event. The mouse and chicken genomes were found to have a similar, conserved GALNT-SAP-HAND organization. The zebrafish has a predicted GALNT gene upstream of the SAP30L gene, and this most likely represents the ancestral chromosome segment because the zebrafish has only one member of the SAP30 family in its genome (see below). In the human genome, the size of the duplicated segment, vectoring the SAP30 family genes between chromosomes 4 and 5, is approximately 400 kb. Comparative analysis of human chromosome 5 [15] has pinpointed this particular 400 kb region as the interchromosomally duplicated segment. Furthermore, our analysis confirms that this region in chromosome 5 is in fact the donor template for the duplication which gave rise to the SAP30 family ~450 Mya, after the Actinopterygii-Sarcopterygii separation [16]. Human chromosome 5 and linkage group 21 (LG21) in the zebrafish have been shown to share most of the conserved syntenies, indicating that these are orthologous chromosomes [17]. Although the genes in these chromosomes were syntenic in the last common ancestor of the zebrafish and human, massive intrachromosomal rearrangements have apparently occurred in the fish and/or mammalian lineages since their divergence. Such rearrangements are known to occur in the SAP30L-harboring human chromosome 5q region [18], which is frequently deleted in myeloid malignancies such as the 5q- syndrome. The synteny between the zebrafish LG21 and human chromosome 5 has been disrupted by intrachromosomal translocations and inversions of chromosome segments. The fact that the GALNT-SAP microsynteny has been preserved between fish and human chromosomes, and between human chromosomes 4 and 5, indicates that these genes may have some kind of cooperative function. Perhaps they are under common regulation or even give rise to chimeric transcripts, which are in fact predicted in the USCS database [19].

### Identification of members of the SAP30 family and conserved regions in the protein

A database of sequences judged to be members of the SAP30 protein family was compiled (Table 1). Altogether, 62 members of the SAP30 family were identified by Psi-Blast searches with the human SAP30L sequence on a non-redundant protein sequence database. SAP30 family sequences were found in a variety of species from Animalia, Plantae and Fungi, as well as in several green algae but not in any chloroplastless protist. In addition, one SAP30 family member in the ambiguous Mycetozoan group was found. In the yeasts, our searches identified SAP30 family members in only two subphyla within the phylum Ascomycota: Saccharomycotina and Pezizomycotina. The third subphylum, Taphrinomycotina seems to have lost SAP30 family proteins during evolution, as we could not find any recognizable sequences from this subphylum, when *Schizosaccharomyces pombe*, as a representative species whose whole genome has been sequenced, was analyzed (from the NCBI database or the *S. pombe *gene database from Sanger Institute). The composition of the Sin3 corepressor complex in *S. pombe *seems to be distinct from that in other eukaryotes as its genome is reported to also lack SAP18 and SDS3 [20], other core members of the complex. According to a comprehensive analysis of yeast evolution [21], Taphrinomycotina is the earliest diverging clade within the phylum Ascomycota, and this divergence is estimated to have occurred ~1140 ± 80 Mya [22]. However, SAP30L was also found in plants and the green algae *Chlamydomonas reinhardtii *(see alignment in additional file 1). Molecular clock analyses indicate that plants separated from the lineage leading to the mycetozoans and fungi about 1580 ± 90 Mya [22] and thus, this is also the estimated age of the SAP30 family.

**Table 1:** SAP30 family proteins used for amino acid sequence alignment.

**Number**	**Name**	**Identifier**	**Organism**	**Taxonomy**	**Description**
1	Hs30	gi|4506783	Homo sapiens	Eu., mt., vt., mam.	SAP30

2	Mam30	gi|109076181	Macaca mulatta	Eu., mt., vt., mam.	PREDICTED: similar to SAP30 isoform 2

3	Pt30	gi|114596884	Pan troglodytes	Eu., mt., vt., mam.	PREDICTED: SAP30 isoform 2

4	Bt30	gi|119896054	Bos taurus	Eu., mt., vt., mam.	PREDICTED: similar to SAP30

5	Mm30	gi|12408290	Mus musculus	Eu., mt., vt., mam.	SAP30

6	Cf30	gi|73993665	Canis familiaris	Eu., mt., vt., mam.	PREDICTED: similar to SAP30

7	Oa30	gi|149412039	Ornithorhynchus anatinus	Eu., mt., vt., mam.	PREDICTED: SAP30

8	Md30	gi|126331237	Monodelphis domestica	Eu., mt., vt., mam.	PREDICTED: similar to SAP30

9	Gg30	gi|118090131	Gallus gallus	Eu., mt., vt., av.	PREDICTED: similar to SAP30

10	Xt30	gi|62860074	Xenopus tropicalis	Eu., mt., vt., amp.	SAP30

11	Xl30	gi|148227208	Xenopus laevis	Eu., mt., vt., amp.	MGC99111 protein

12	Hs30L	gi|74734226	Homo sapiens	Eu., mt., vt., mam.	SAP30L, NS4ATP2, FLJ11526

13	Mam30L	gi|109079479	Macaca mulatta	Eu., mt., vt., mam.	Predicted: similar to SAP30L

14	Bt30L	gi|119923830	Bos taurus	Eu., mt., vt., mam.	Predicted: hypotethical protein

15	Mm30L	gi|124487193	Mus musculus	Eu., mt., vt., mam.	SAP30L

16	Rn30L	gi|109490760	Rattus norvegicus	Eu., mt., vt., mam.	PREDICTED: similar to SAP30L

17	Gg30L	gi|118097434	Gallus gallus	Eu., mt., vt., av.	Predicted: hypotethical protein

18	Xt30L	gi|62858467	Xenopus tropicalis	Eu., mt., vt., amp.	hypothetical protein LOC549895

19	Xl30L	gi|160358663	Xenopus laevis	Eu., mt., vt., amp.	SAP30L-A

20	Dr30L	gi|47550711	Danio rerio	Eu., mt., vt., acti.	SAP30L

21	Tn30L	gi|47221378	Tetraodon nigroviridis	Eu., mt., vt., acti.	unnamed protein product

22	Aa30L	gi|157112936	Aedes aegypti	Eu., mt., art., ins.	SAP30

23	Ag30L	gi|118794370	Anopheles gambiae str. PEST	Eu., mt., art., ins.	AGAP001654-PA

24	Am30L	gi|66509501	Apis mellifera	Eu., mt., art., ins.	PREDICTED: similar to CG4756-PA

25	Dp30L	gi|125983642	Drosophila pseudoobscura	Eu., mt., art., ins.	GA18408-PA SAP30

26	Dm30L	gi|18859859	Drosophila melanogaster	Eu., mt., art., ins.	CG4756-PA SAP30

27	Tc30L	gi|91080611	Tribolium castaneum	Eu., mt., art., ins.	PREDICTED: similar to CG4756-PA

28	Sp30L	gi|115610671	Strongylocentrotus purpuratus	Eu., mt., ech	PREDICTED: similar to Sap30-like

29	Nv30L	gi|156369622	Nematostella vectensis	Eu., mt., cnid.	predicted protein

30	Dd30L	gi|66810369	Dictyostelium discoideum AX4	Eu., myc.	hypothetical protein DDBDRAFT_0185724

31	Vv30L	gi|157335386	Vitis vinifera	Eu., virid., strept.	unnamed protein product

32	Vp30L	gi|85070180	Vitis pseudoreticulata	Eu., virid., strept.	unknown

33	At30L	gi|18394724	Arabidopsis thaliana	Eu., virid., strept.	unknown protein

34	At30L-B	gi|145327243	Arabidopsis thaliana	Eu., virid., strept.	unknown protein

35	Osj30L	gi|78708341	Oryza sativa (japonica cultivar-group)	Eu., virid., strept.	expressed protein

36	Osj30L-B	gi|115457076	Oryza sativa (japonica cultivar-group)	Eu., virid., strept.	Os04g0166600

37	Cr30L	gi|159464042	Chlamydomonas reinhardtii	Eu., virid., chlor.	hypothetical protein CHLREDRAFT_190150

38	Ol30L	gi|145350235	Ostreococcus lucimarinus CCE9901	Eu., virid., chlor.	predicted protein

39	Ot30L	gi|116059598	Ostreococcus tauri	Eu., virid., chlor.	unnamed protein product

40	Yl30L	gi|50556448	Yarrowia lipolytica	Eu., Fungi, sacch.	hypothetical protein

41	Asg30L	gi|45190881	Ashbya gossypii ATCC 10895	Eu., Fungi, sacch.	AER278Wp

42	Kl30L	gi|50308899	Kluyveromyces lactis	Eu., Fungi, sacch.	unnamed protein product

43	Cg30L	gi|50288935	Candida glabrata	Eu., Fungi, sacch.	unnamed protein product

44	Vap30L	gi|156845457	Vanderwaltozyma polyspora DSM 70294	Eu., Fungi, sacch.	hypothetical protein Kpol_541p4

45	Ps30L	gi|126136507	Pichia stipitis CBS 6054	Eu., Fungi, sacch.	predicted protein

46	Sc30L	gi|6323919	Saccharomyces cerevisiae	Eu., Fungi, sacch.	SAP30

47	Dh30L	gi|50425161	Debaryomyces hansenii CBS767	Eu., Fungi, sacch.	hypothetical protein DEHA0F20284g

48	Pg30L	gi|146421845	Pichia guilliermondii ATCC 6260	Eu., Fungi, sacch.	hypothetical protein PGUG_00243

49	Ca30L	gi|68489492	Candida albicans SC5314	Eu., Fungi, sacch.	putative SAP30

50	Le30L	gi|149237879	Lodderomyces elongisporus NRRL YB-4239	Eu., Fungi, sacch.	conserved hypothetical protein

51	Bf30L	gi|154298394	Botryotinia fuckeliana B05.10	Eu., Fungi, pez.	hypothetical protein BC1G_11652

52	Ci30L	gi|119178679	Coccidioides immitis RS	Eu., Fungi, pez.	hypothetical protein CIMG_08147

53	Chg30L	gi|116196544	Chaetomium globosum CBS 148.51	Eu., Fungi, pez.	hypothetical protein CHGG_04870

54	Ac30L	gi|121715712	Aspergillus clavatus NRRL 1	Eu., Fungi, pez.	conserved hypothetical protein

55	Af30L	gi|71001656	Aspergillus fumigatus Af293	Eu., Fungi, pez.	conserved hypothetical protein

56	An30L	gi|145232103	Aspergillus niger	Eu., Fungi, pez.	hypothetical protein An02g03790

57	Scc30L	gi|156045101	Sclerotinia sclerotiorum 1980	Eu., Fungi, pez.	hypothetical protein SS1G_09739

58	Nf30L	gi|119481227	Neosartorya fischeri NRRL 181	Eu., Fungi, pez.	conserved hypothetical protein

59	Nc30L	gi|85105620	Neurospora crassa OR74A	Eu., Fungi, pez.	hypothetical protein

60	Ao30L	gi|83769778	Aspergillus oryzae	Eu., Fungi, pez.	unnamed protein product

61	Asn30L	gi|67540052	Aspergillus nidulans FGSC A4	Eu., Fungi, pez.	hypothetical protein AN6196.2

62	Pn30L	gi|160703739	Phaeosphaeria nodorum SN15	Eu., Fungi, pez.	hypothetical protein SNOG_12725

Note that the S. cerevisiae SAP30 is judged to be SAP30L (see data presented below) and named as such (Sc30L) for coherence. acti. = Actinopterygii, amp. = Amphibia, art. = Arthropoda, eu. = Eukaryota, ins. = Insecta, invt = Invertebrata, mam. = Mammalia, mt = Metazoa, vt = Vertebrata, av. = Aves, ech. = Echinodermata, cnid. = Cnidaria, myc. = Mycetozoa, virid. = Viridiplantae, chlor. = Chlorophyta, strept. = Streptophyta, sacch. = Saccharomycotina, pez. = Pezizomycotina.

**Figure 2:**
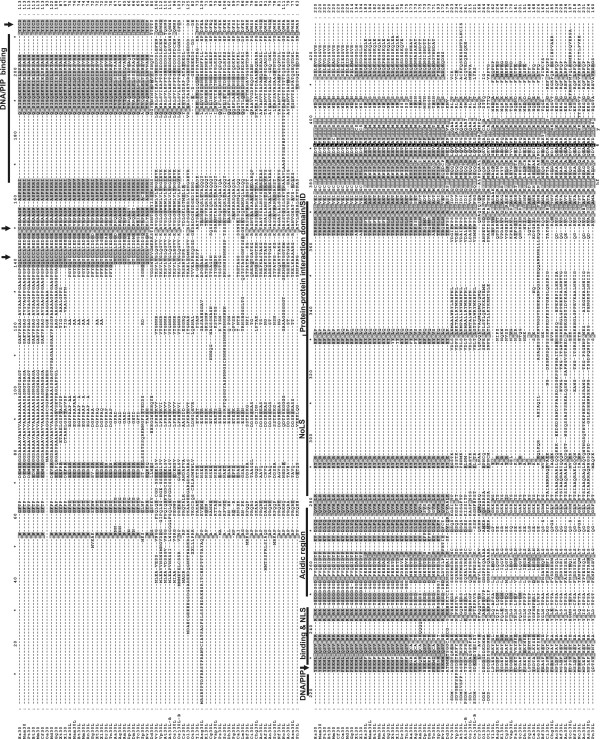
**Amino acid sequence alignment of the members of the SAP30 family**. Naming of the sequences is presented in Table 1. The residues in the alignment are shaded light grey, grey, or black to indicate shared identity at 40%, 70% and 100%, respectively. The arrows indicate the zinc coordinating residues. PIP = Phosphatidyl Inositol Phosphate, NLS = Nuclear localization signal, Acidic region = a central region contributing to histone/nucleosome binding, NoLS = Nucleolar localization signal, SID = Sin3 interacting domain.

Multiple Clustal W alignment of sequences of the SAP30 family identified a highly conserved region in the C-terminus (Figure 2), which consists mainly of aliphatic (I, L, V), aromatic (F, Y, W, H) and charged (H, K, R, D, E) residues. Moreover, when the alignment is examined according to the physiochemical properties of the amino acids, this region shows 100% conservation in the nine C-terminal residues. The consensus sequence of this conserved C-terminal motif is [hydrophobic]-x(2)-[hydrophobic]-[hydrophobic]-x(4)-[hydrophobic] -x-[amphoteric]-x(2)-[hydrophobic]-[aliphatic]-x(2)-[hydrophobic]-[hydrophobic].

The Clustal W alignment of SAP30/SAP30L sequences shown in Figure 2 also revealed that the nucleolar localization signal (NoLS) [8], which consists of basic amino acids is quite conserved among the species studied. Although there is no striking co-aligning NoLS in yeast and plants sequences, they contain polybasic region preceding C-terminal motif. As a conclusion, the C-terminal domain, NoLS motif and the Sin3-interacting domain (SID) represent the most ancient region in proteins of the SAP30 family, and this domain evidently appeared early in the evolution of this family. Despite the apparent lack of a co-aligning NoLS in yeasts, the reported functions of SAP30 in rDNA transcription [9,10] and ribosome biogenesis [11] suggest that SAP30 is targeted to the nucleolus, and fulfills these functions in yeasts as well.

### Conserved domain structure in proteins of the SAP30 family within animals

The alignment of animal SAP30 and SAP30L sequences revealed several conserved regions in these proteins (Figure 2 and Additional file 2). One of these is the N-terminal zinc-dependent module, in which all four zinc-coordinating residues (CCCH) [12] are strictly conserved. The distances between these zinc-coordinating residues are highly conserved as C-×(8)-C-×(35)-C-×(2)-H, suggesting that they are critical for proper folding of the zinc-binding module. The amino acids at the DNA-binding interface in the loop region are also well conserved, and show mainly conservative substitutions consisting of polar and basic residues. The DNA/PIP-binding domain [12], which constitutes the NLS motif [7] and comprises the polybasic region adjacent to the zinc-binding module, is also well conserved, as are the NoLS motif, SID domain and the acidic central region that contributes to histone and nucleosome binding [7,8,12]. The high degree of conservation indicates that all these modules are probably important for the function of the SAP30 family proteins.

### Phylogenetic analysis and timing of the SAP30L gene duplication

**Figure 3:**
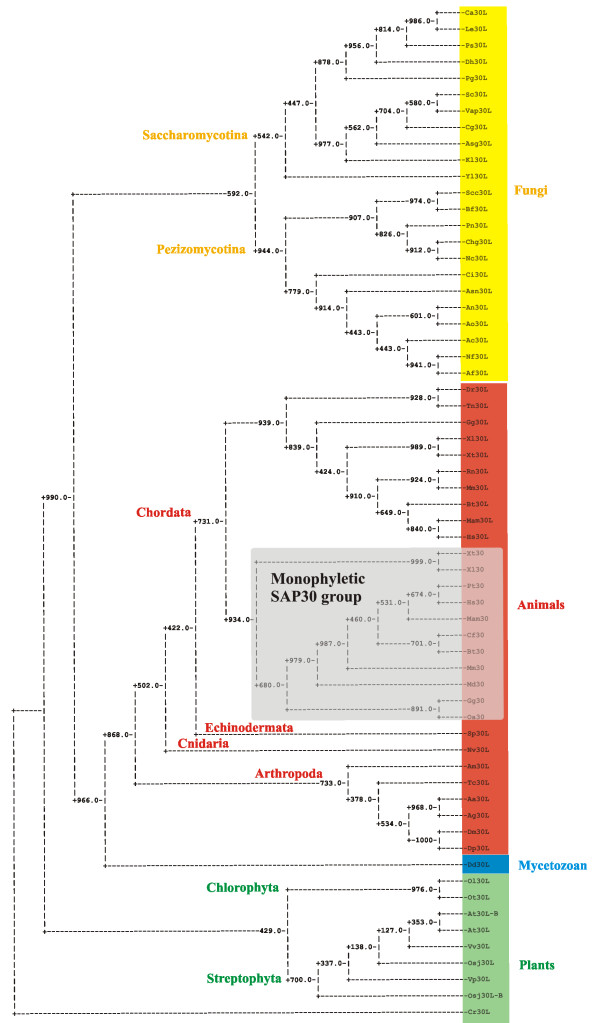
**A phylogenetic tree of the SAP30 protein family**. The tree was derived by a neighbor-joining distance analysis (the parsimony and likelihood trees are presented in Additional files 4 and 5). The statistical reliability of the inferred tree topology was assessed by the jackknife test, and the values are shown at each node as a percentage calculated from 1000 data sets.

Phylogenetic trees were generated from the Clustal W alignment (Figure 2) of the SAP30 protein sequences presented in Table 1 using the distance, parsimony and likelihood methods. Statistical confidence was measured by Jackknife analysis with 1000 "delete-half jackknife" data sets except in the likelihood method, in which case only 100 data sets were measured due to constraints imposed by computation time. All three methods gave trees with congruent topologies, the main discrepancies being the varying positions of the single representatives from the Echinodermata, Cnidaria and Mycetozoa. A reliable positioning of these sequences would probably require more data from these taxa, and prefererably from the intermediate taxa as well, but the content of the current databases does not allow this. The extensive sequence divergence observed within the yeasts (Additional file 3) may also explain the non-monophyly of the Ascomycota in the both the parsimony and the likelihood trees. In the distance tree (Figure 3), as well as in the parsimony and likelihood trees (Additional files 4 and 5), SAP30 proteins clearly fall into one group (with Jackknife percentage values of 93.4%, 99.8% and 88% for the distance, parsimony and likelihood methods, respectively). This strongly supports a single origin for the SAP30 protein family. The presence of SAP30L and the absence of SAP30 in the fish (*Danio rerio *and *Tetraodon nigroviridis*) genomes suggests that the SAP30 gene originated from the ancestral SAP30L gene by duplication of a chromosome segment after the divergence of fishes (Actinopterygii, ray-finned fishes) but before the divergence of amphibians which belong to the Sarcopterygii (lobe-finned fishes). In fact, according to an analysis by Friedman et al. 77.7% of the interchromosomal duplication events that can be seen in the human genome have occurred prior to the amniote-amphibian separation [14]. Careful inspection of the animal SAP30/SAP30L sequences (Additional file 2) reveals that many amino acid substitutions are characteristic for either SAP30 or SAP30L (apomorphic), and therefore carry phylogenetic information about the duplication of the SAP30L gene.

### Functional divergence of the paralogous SAP30 and SAP30L genes

**Figure 4:**
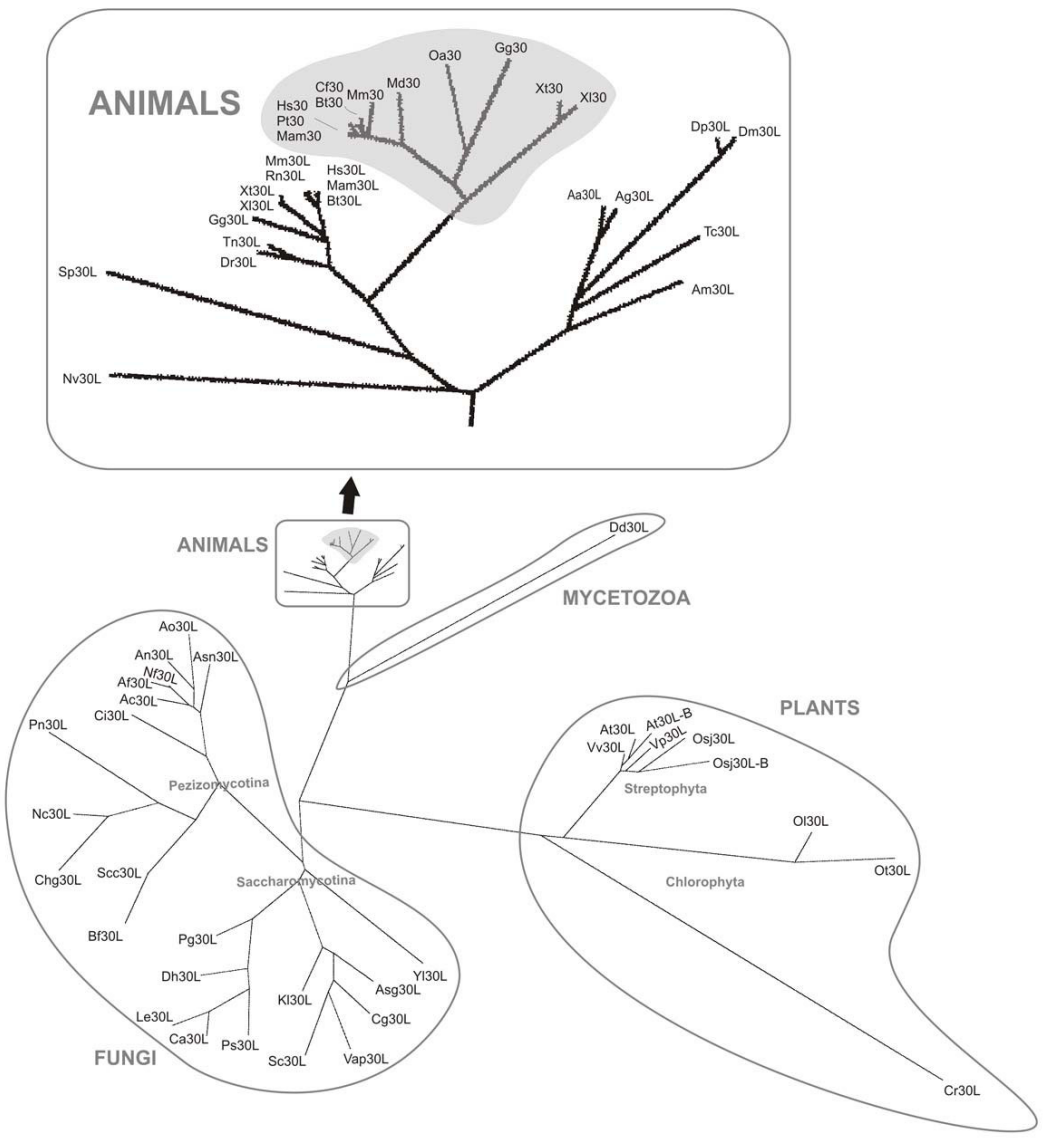
**A phylogenetic tree of the SAP30 family in which branch lengths are proportional to the extent of sequence divergence**. The black arrow points to the tip of the animal branch, which is shown magnified in the lower left corner. The dispersed, monophyletic tetrapodan/sarcopterygian SAP30 group is shaded.

It is noteworthy that the tetrapodan SAP30 orthologs from frogs to humans (Sarcopterygii) are much more dispersed in the distance tree than are the SAP30L orthologs in the corresponding species (Figure 4). This is also evident in the alignment of animal SAP30/SAP30L sequences (Additional file 2). The sarcopterygian SAP30 proteins contain more amino acid substitutions, and many more deletions and insertions, than the SAP30L proteins. In fact, the divergence and amino acid identity values for SAP30 protein sequences between *Homo sapiens *and *Xenopus tropicalis *are 27.2% and 74.2%, respectively, whereas the corresponding values for SAP30L are 9.5% and 89%. It seems that since their divergence by segmental duplication from a common ancestor, the evolutionary rate in SAP30 proteins has been much higher than in SAP30L proteins. This is what is thought to occur more generally in duplicated genes, where the new copy will evolve unencumbered by the selective constraints imposed on its progenitor [23]. Furthermore, the evolutionary rate of amino acid substitution may increase and functional divergence may take place at the early stage of evolution after separation [24]. This is followed by the late stage, in which purifying selection plays a major role in maintaining related, but distinct functions. This has allowed SAP30 to gain more length, mainly by microsatellite expansion near its N-terminus. It is not known if this has produced "gene innovation" (i.e. addition of a new functional domain) to SAP30, because the function of this N-terminal extension is currently unknown.

**Figure 5:**
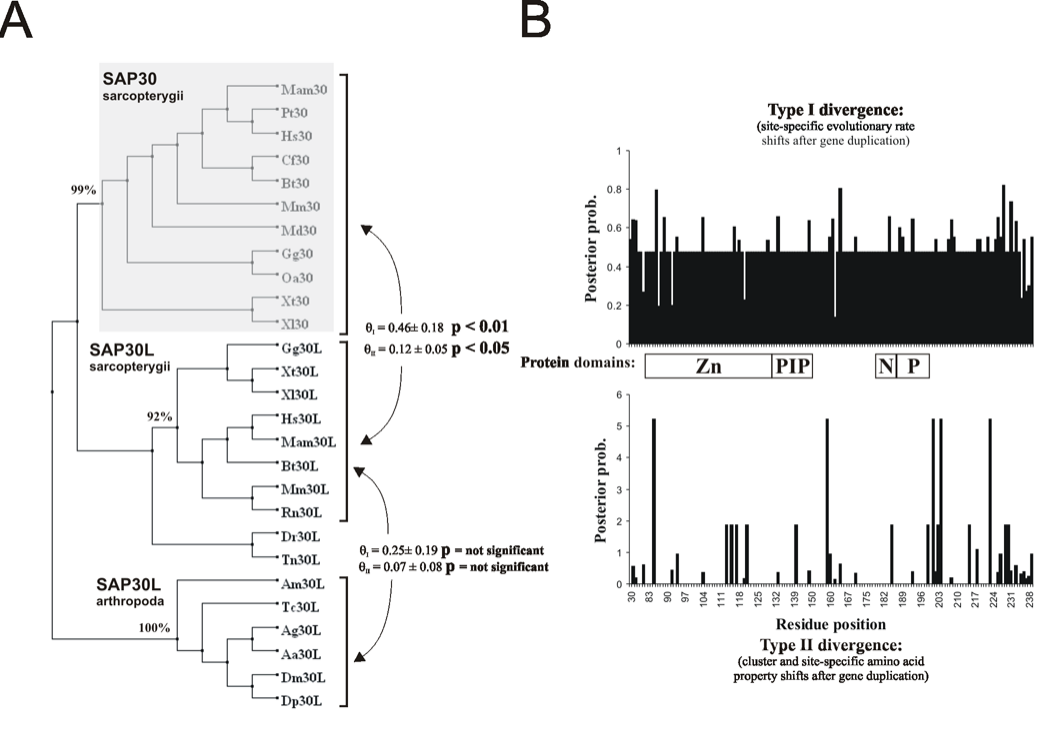
**Functional divergence between SAP30 and SAP30L**. a) A neighbor-joining tree of the tetrapodan/sarcopterygian SAP30 and SAP30L, and the arthropodan SAP30L, showing the jackknife values at the nodes. The monophyletic tetrapodan/sarcopterygian SAP30 cluster is shaded. The curved arrows indicate comparisons for type-I (θ_I_) and type-II (θ_II_) functional divergence, and statistically significant p-values are indicated. b) Posterior probability plot of amino acid positions indicative of type-I or type-II functional divergence. The conserved domains are depicted in the white boxes between the plots. Zn = zinc dependent module, PIP = monophosphoinositide binding motif, N = nucleolar lozalization signal, P = protein-protein interaction domain.

Since the cluster-specific residues between SAP30 and SAP30L in the sarcopterygian clusters were prominent in the alignment, we tested whether these residues are functionally relevant. Functional significance is highly correlated with evolutionary conservation [25]. If a particular amino acid site is variable in both clusters, it is unlikely to have any major functional role in either paralog. Conversely, conservation of an amino acid in one cluster and lack of conservation in a sister cluster is assumed to contribute to functional differences between the paralogs. This site-specific shift in the evolutionary rate between clusters is known as type-I functional divergence [26]. In type-II functional divergence, a particular site is conserved in both clusters but the physicochemical property of the amino acid is different between the clusters [27]. To test if the cluster-specific residues in SAP30 and SAP30L are indicative of type-I divergence, we estimated the coefficient of functional divergence (θ), which measures the difference in the evolutionary rate at amino acid sites between gene clusters. Rejection of the null hypothesis (θ = 0) is strong evidence for altered functional constraints after gene duplication (or speciation) [28]. We found significant evidence for type-I divergence in the comparison between sarcopterygian SAP30 and SAP30L clusters (θ_I _= 0.46 ± 0.18, p < 0.01 Figure 5a), but not in the control comparison between sarcopterygian and arthropodan SAP30L clusters (θ_I _= 0.25 ± 0.19, p > 0.19), which reflects the situation before the gene duplication. Similarly, significant type-II functional divergence was detected only between SAP30 and SAP30L clusters (θ_II _= 0.12 ± 0.05, p < 0.05), whereas it was undetectable before the gene duplication (θ_II _= 0.07 ± 0.08, p > 0.37). To put these findings into perspective, the human SAP30 and SAP30L share 70% amino acid identity, whereas the human SAP30L and the *Drosophila melanogaster *SAP30L share only 50% identity. This latter comparison clearly shows that it is mostly the neutral amino acid sites with no functional role that are variable, whereas after the gene duplication (i.e. the emergence of SAP30), functional divergence has taken place.

To conclude, after the sarcopterygian radiation around 450 Mya, the duplicated SAP30 has diverged functionally from the ancestral SAP30L. In contrast, evolutionary constraints have kept SAP30L functionally unchanged for ~1000 My, since the separation of arthropods and sarcopterygians [29]. In spite of considerable divergence in sequence, only functionally insignificant changes constitute the sequence differences in SAP30L between these two clades. This presumably reflects the fact that purifying selection has acted to conserve SAP30L.

The site-specific profile for the amino acid residues deemed responsible for type-I and type-II functional divergence (Figure 5b) show that most of the functional divergence is found in the C-terminal region and between the experimentally identified domains. However, previous experiments have shown that: i) the repression capacity of SAP30 is only half of that of SAP30L, ii) SAP30L is able to self-oligomerize *in vivo *whereas SAP30 is not, and iii) SAP30L is more concentrated in the nucleolus than SAP30 in transfection experiments [8]. These biochemical data, together with the molecular evolutionary analysis described here, suggest that the original functions are executed by SAP30L, but in SAP30 these functions are abandoned or suppressed.

### The functional divergence between SAP30 and SAP30L is due to differences in their association with the nuclear matrix

**Figure 6:**
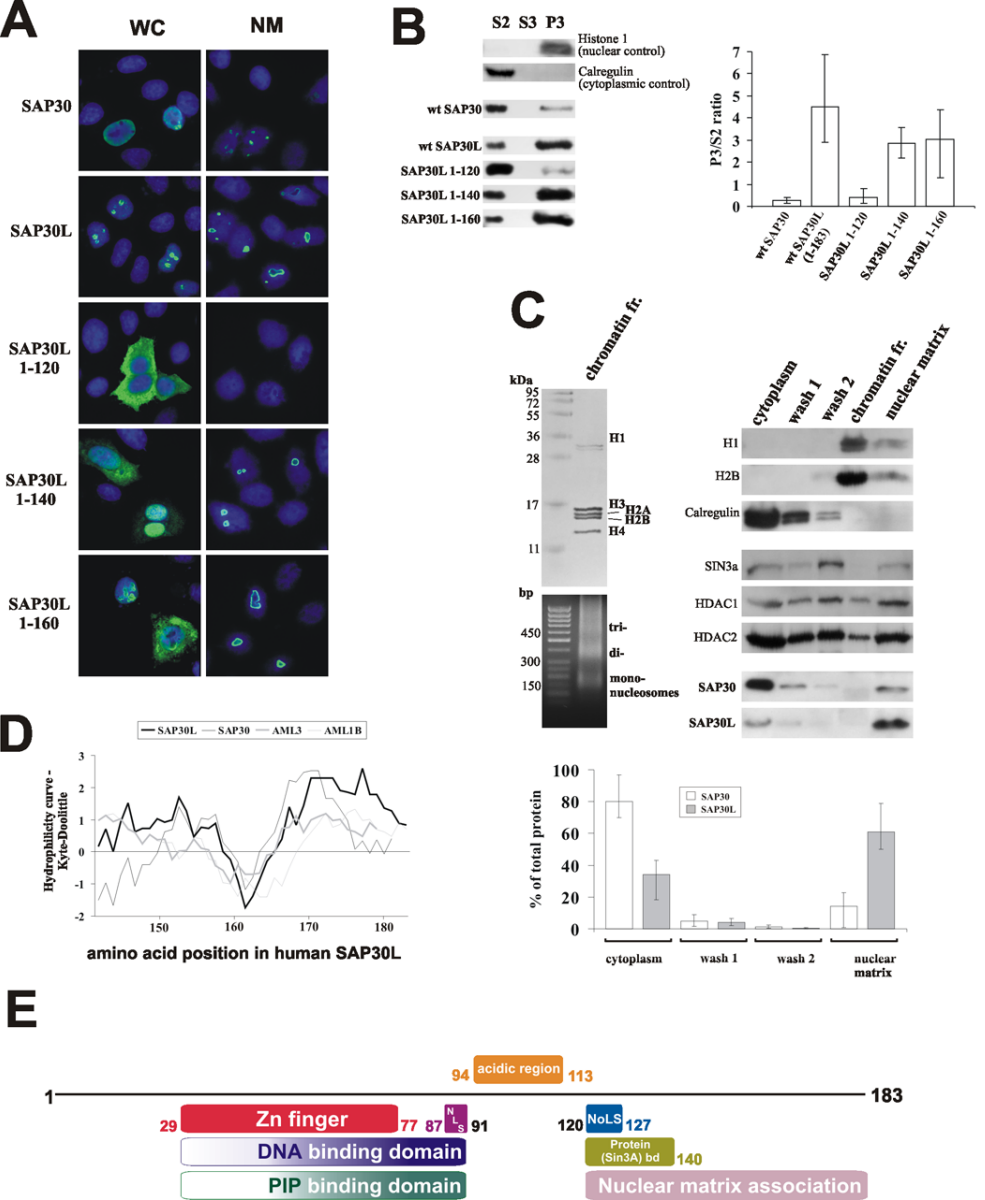
**A nuclear matrix association sequence consists of the nucleolar localization signal and the conserved C-terminus**. a) Hela cells were transfected with the indicated, myc-tagged constructs and the nuclear matrix was prepared. NM, nuclear matrix preparation; WC, whole cell. Subsequently the cells were stained with an antibody against the myc tag, mounted in DAPI and photographed on a confocal microscope. b) HEK293T cells were transfected with the indicated constructs, fractionated into subcellular fractions, and immunoblotted with the antibodies as indicated. S2, S3 and P3 correspond to the cytoplasmic soluble, nuclear soluble and nuclear insoluble (chromatin and nuclear matrix) fractions, respectively. The data from three independent experiments are illustrated as histograms in which the bars represent the range of band intensities measured with a densitometer. c) HEK293T cells were transfected with myc-tagged SAP30 and SAP30L proteins, and nucleosomes were isolated. In the left upper panel, a Coomassie-stained gel shows release of histones, and an agarose gel (left lower panel) shows the accompanying release of nucleosomal DNA from the nucleus after treatment with micrococcal nuclease. The proteins from each step of nucleosome isolation were analysed on the immunoblot shown in the right panel. The data from the three independent experiments are illustrated in the histograms, as in (b). d) A Kyte-Doolittle Hydrophilicity plot of the nuclear matrix association sequences from proteins of the SAP30 and AML [43] families. e) A schematic representation of the domains identified in SAP30L. NLS, nuclear localization signal; NoLS, nucleolar localization signal; Protein bd, the protein-binding domain and nuclear matrix association sequence identified in this study. The numbers indicate amino acid positions. The color gradients depict more strongly interacting regions in darker colors. The zinc finger is necessary for proper presentation of these regions to DNA or phosphoinositides.

Although the C-terminal region is the most conserved part in proteins of the SAP30 family, considerable type-I and type-II functional divergence has occurred in this region after the separation of the SAP30L and SAP30 genes (Figure 5b). Our previous subcellular fractionation experiments showed that nuclear retention of SAP30L is achieved by interaction with DNA through the N-terminal domain [12]. We also demonstrated that the C-terminus has a role in nuclear retention, because C-terminally truncated mutants of SAP30L leaked to the cytoplasm in transfection studies [8,12]. We therefore asked whether the C-terminal region constitutes a nuclear matrix association sequence. When myc-tagged constructs of wild type (wt) SAP30 and SAP30L were transfected into HeLa cells and the nuclear matrix was isolated, we noticed that staining of the perinucleolar ring was resistant to Triton-X and DNAse I treatments, indicating that the proteins remained attached to the nuclear matrix in the perinucleolar ring region (Figure 6a). SAP30L1-120 was completely soluble, while the 1–140 and 1–160 versions showed some attachment to the nuclear matrix (Figure 6a). Intriguingly, SAP30L seemed to be bound more tightly than SAP30, suggesting that SAP30 has accumulated mutations that hinder its association with the nuclear matrix. In order to gain more quantitative data, we performed subcellular fractionation experiments and found that SAP30 was considerably more soluble than SAP30L, which accumulated in the nuclear matrix/chromatin fractions. The 1–120 mutant of SAP30L showed markedly reduced accumulation in the nuclear matrix/chromatin fraction, and the 1–140 and 1–160 mutants were also more soluble than wt SAP30L (Figure 6b), indicating that an intact C-terminus is necessary for the association with the nuclear matrix (see Figure 6e for a schematic representation of the domains identified in SAP30L). Since the nuclear matrix participates in gene transcription [30] and repression [31], the impaired association of the 1–140 version of SAP30L with the nuclear matrix could explain its previously observed, reduced repression activity [8].

We recently showed that the N-terminal zinc-dependent module and the following hydrophobic region together with polybasic region/NLS are needed for DNA binding *in vitro *and chromatin association *in vivo *[12]. As shown in Figure 6c (left panel), solubilization of chromatin with micrococcal nuclease does not detach wt SAP30 or wt SAP30L from the nuclear matrix. Their attachment is dependent on an intact C-terminus, which thus constitutes a nuclear matrix association sequence (Figure 6c, right panel). The association of SAP30 with the nuclear matrix seems to be weaker than that of SAP30L. Alternatively, SAP30 may possess a less effective NLS or NoLS and/or a more effective nuclear export signal (NES). Interestingly, Sin3A has also been reported to associate with the nuclear matrix [32], and we used it as a control nuclear matrix protein. Taken together, these findings show that proteins of the SAP30 family are able to interact with co-repressors (e.g. Sin3, N-CoR), associate with the nuclear matrix, and bind DNA, and therefore possess many characteristics typical of nuclear scaffolding proteins [33]. A well studied example of nuclear scaffolding proteins is provided by members of the RUNX family of transcription factors, which are tissue-specific regulatory proteins involved in the control of hematopoiesis (Runx1/AML1), osteogenesis (Runx2/AML3), and differentiation of neural and gastrointestinal cells (Runx3/AML2) [34]. Their N-terminal parts bind specific DNA sequences, whereas the C-terminal domains interact with coregulatory factors and associate with the nuclear matrix [34], a domain organization similar to that in proteins of the SAP30 family. In addition, the subcellular localization of proteins of the two families also bears similarities, as Runx proteins are focally localized within the nucleus and some of them are actually found in the nucleolus [35]. Interestingly, the nuclear matrix association sequence in both protein families is comprised of a stretch of hydrophobic residues flanked by hydrophilic residues (Figure 6d).

It is now widely accepted that in higher organisms such as mammals, a particular function is often assigned to a gene family rather than to a single gene. Many gene families are thought to have originated by gene duplication at an evolutionary stage when most vertebrates were still aquatic [36]. The members of a gene family perform the same or similar function, but in slightly different and overlapping ways. In the case of the SAP30 family, these subtle differences may be exploited during ontogeny, given the crucial role reserved for the Sin3A complex in embryogenesis [1].

## Conclusion

In this report, we have described the molecular evolution of the SAP30 protein family and its genesis from a single chromosome segment duplication event. Our analyses indicate that the ancestral SAP30L protein is conserved in animals, plants, fungi and some chloroplast-containing protists. We have identified many new members of the SAP30 family from different species and a conserved C-terminal domain which is responsible for association with the nuclear matrix. The phylogenetic and biochemical analyses have uncovered functional divergence between SAP30 and SAP30L in the domain that associates with the nuclear matrix. These data will facilitate further studies on the functional role of proteins of the SAP30 family in the Sin3-HDAC corepressor complex, and possibly other complexes as well.

## Methods

### Protein sequence searches, gene locus data retrieval and multiple sequence alignments

Protein Psi-Blast [37] searches with the full length human SAP30L sequence were performed at the NCBI Web site  on the non-redundant protein sequence database available on December 3, 2007. After six rounds of iteration, SAP30 and SAP30L orthologs below an E-value of 0.005 (except for *Phaeosphaeria nodorum*, for which the E-value was 0.011) were selected from metazoa, plants and fungi, and all redundant sequences were excluded. SAP30 and SAP30L proteins are encoded in four exons, and variable usage of these exons is reported to yield multiple splicing variants [38]. It is also predicted that the longer SAP30 and SAP30L cDNAs are composed of additional spliced-in, upstream exons. These predicted additional exons (*Rattus norvegicus *SAP30L, gi|109490760) were excluded from our analyses, all of which were done on protein sequences that contained the four complete exons, for the sake of clarity. All sequences were collected in FASTA format for further analysis as shown in Table 1. The identification and naming of the protein sequences as either SAP30 or SAP30L is based on the phylogenetic analyses shown in Figures 3 and 4. The SAP30 and SAP30L sequences were aligned using the MegAlign 5.06^© ^program (DNASTAR Inc) with Clustal V [39] or W [40] at default settings. The alignments were then shaded using the multiple sequence alignment editor GENEDOC . Gene locus data were retrieved from the NCBI Map viewer .

### Phylogenetic analysis and detection of functional divergence

PHYLIP version 3.67 [41] was used for the phylogenetic analyses. Distance, parsimony and likelihood analyses were performed using the protein alignment as input. Jackknife values were obtained using SEQBOOT and creating 1000 or 100 "delete-half jackknife" data sets. The distance analysis was performed by using PROTDIST and subsequently NEIGHBOR with standard parameters, and the parsimony analysis was performed using PROTPARS with standard parameters. The Likelihood analysis was performed by using PROML with standard parameters. In all cases, the "M" option for the analysis of multiple data sets created with SEQBOOT was invoked.

We used DIVERGE version 2.0 [42] for detecting type-I [26] and type-II [27] functional divergence. Clustal W alignments of the arthropodan and sarcopterygian clades for SAP30L and the sarcopterygian clade for SAP30 were created, and a distance analysis with 100 "delete-half jackknife" data set tests was performed using PHYLIP as described above. It should be noted that we used tetrapod sequences as representatives of the sarcopterygian clade due to the lack of sequence data from the Dipnoi (lungfish) and Coelacanthimorpha infra- and subclasses. The alignment and the neighborjoining tree were used as input for the functional divergence analyses. P-values were derived from the θ and standard error values using the Z-score.

### Cell culture and transfections

Human embryonic kidney epithelial cells (HEK293T) were cultured in DMEM (Gibco) containing 5% fetal bovine serum, 1 mM sodium pyruvate, 50 μg/ml uridine, penicillin and streptomycin. HeLa cells were cultured in RPMI1640 (Gibco) supplemented with 10% fetal bovine serum, L-glutamine, penicillin and streptomycin. DNA was transfected using FuGENE 6 (for HEK293T cells) or FuGENE HD (for HeLa cells) reagents (Roche) according to the manufacturer's protocol.

### Preparation and staining of the nuclear matrix

Nuclear matrix preparations were done as described [43]. Subsequently the cells were fixed and stained as described previously [8].

### Preparation of the chromatin-enriched fraction and nucleosomes

Isolation of chromatin and the nuclear matrix, and subcellular fractionation were performed as described previously [44]. Nucleosomes were prepared as described in [45].

## Authors' contributions

KMV designed the study, carried out the phylogenetic analyses, performed the laboratory work and wrote the manuscript. TYKH was involved in the conception of the study, and participated in the interpretation of the data and writing of the manuscript. MM participated in the coordination of the project and helped to draft the manuscript. OL contributed to the conception and design of the study and helped to write the manuscript. All authors have read and approved the final manuscript.

## Supplementary Material

Additional file 1 **Clustal V alignment of the SAP30 family members in plants.** Click here for file

Additional file 2 **Clustal V alignment of the SAP30 family members in animals**. The arrows indicate the zinc coordinating residues. Red arrows and green boxes indicate the locations of secondary structural elements as deduced from the solution structure [13]. Click here for file

Additional file 3 **Clustal W alignment of the SAP30 family members in yeasts.** Click here for file

Additional file 4 **Maximum parsimony phylogenetic tree of the SAP30 protein family**. The statistical reliability of the inferred tree topologies was assessed by the jackknife test. The jackknife values from 1000 data sets are shown at each node. The monophyletic tetrapodan/sarcopterygian SAP30 group is shaded. Click here for file

Additional file 5 **Maximum likelihood phylogenetic tree of the SAP30 protein family**. The statistical reliability of the inferred tree topologies was assessed by the jackknife test. The jackknife values from 100 data sets are shown at each node. The monophyletic tetrapodan/sarcopterygian SAP30 group is shaded. Click here for file
