# Supplementary figures and images for: Phylogenetic analysis of the SAP30 family of transcriptional regulators reveals functional divergence in the domain that binds the nuclear matrix
Source: BMC Evol Biol. 2009 Jun 30;9:149. doi: 10.1186/1471-2148-9-149 (PMC2711940; doi:10.1186/1471-2148-9-149)

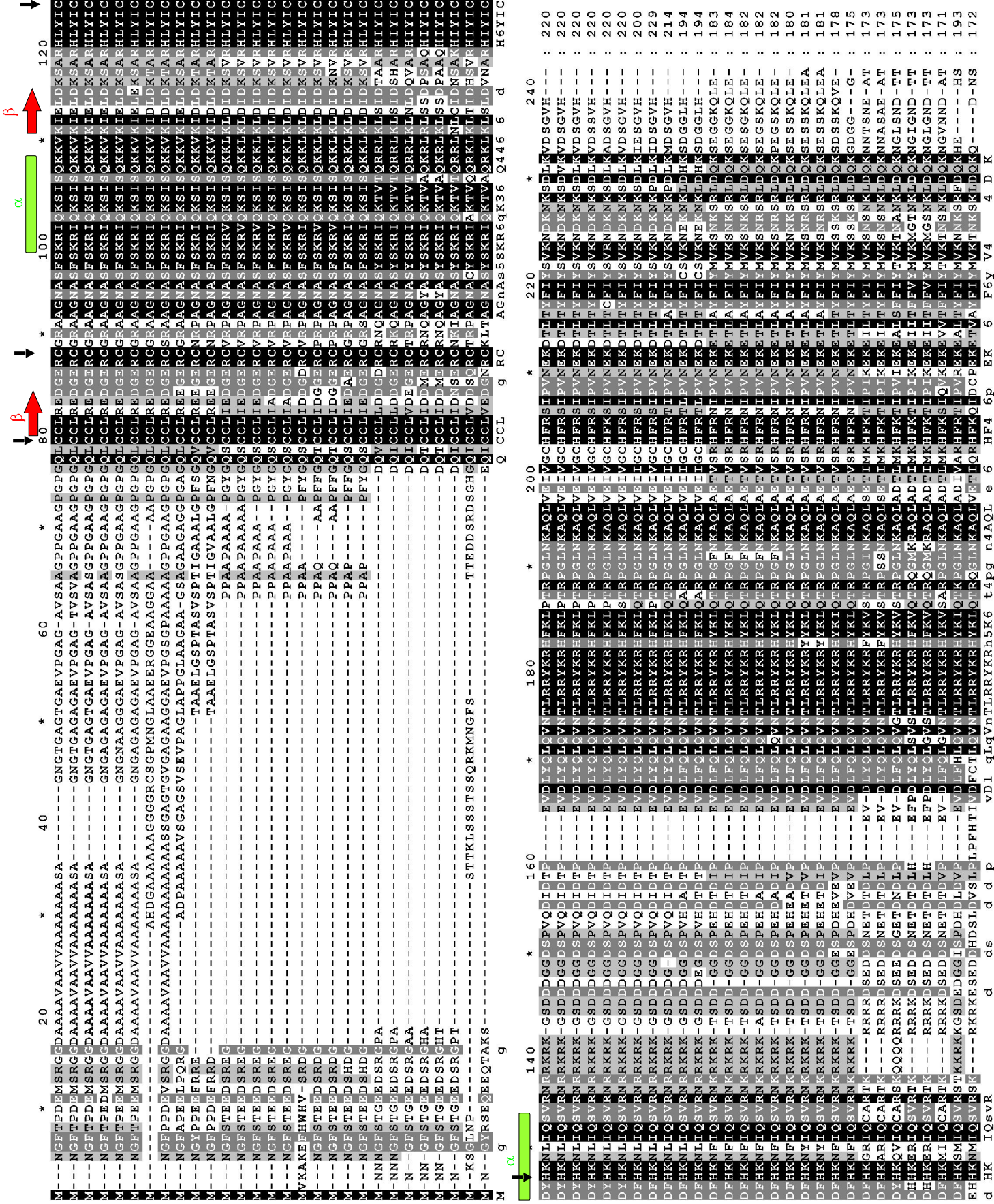

Supplement: Additional file 2 — Clustal V alignment of the SAP30 family members in animals. The arrows indicate the zinc coordinating residues. Red arrows and green boxes indicate the locations of secondary structural elements as deduced from the solution structure [13]. [file 1471-2148-9-149-S2.pdf]

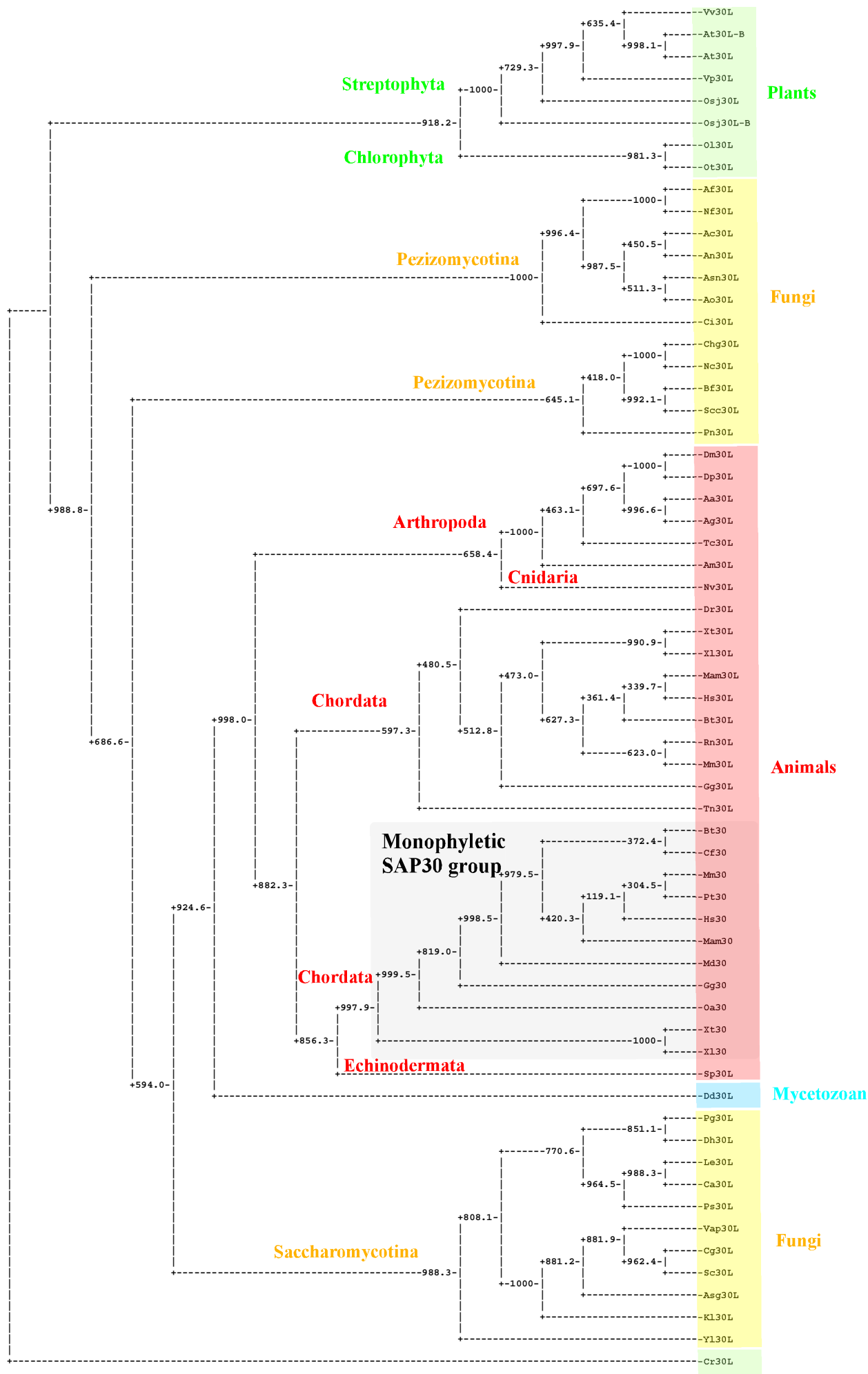

Supplement: Additional file 4 — Maximum parsimony phylogenetic tree of the SAP30 protein family. The statistical reliability of the inferred tree topologies was assessed by the jackknife test. The jackknife values from 1000 data sets are shown at each node. The monophyletic tetrapodan/sarcopterygian SAP30 group is shaded. [file 1471-2148-9-149-S4.pdf]

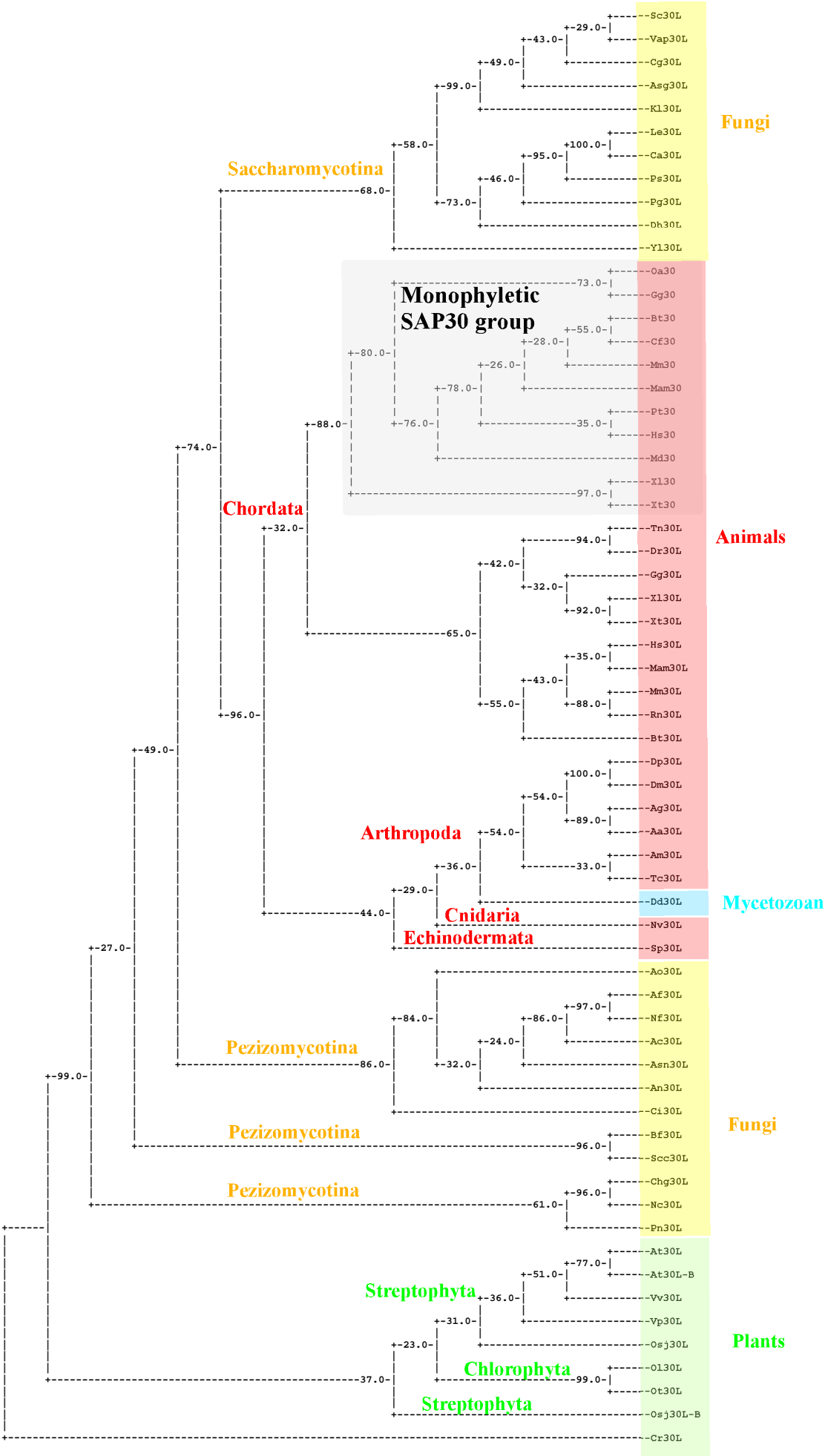

Supplement: Additional file 5 — Maximum likelihood phylogenetic tree of the SAP30 protein family. The statistical reliability of the inferred tree topologies was assessed by the jackknife test. The jackknife values from 100 data sets are shown at each node. The monophyletic tetrapodan/sarcopterygian SAP30 group is shaded. [file 1471-2148-9-149-S5.pdf]
